# The impact of mechanical ventilation on long-term survival influences definitions of persistent critical illness

**DOI:** 10.62675/2965-2774.20250388

**Published:** 2025-07-15

**Authors:** Paula Pinheiro Berto, Cassiano Teixeira, Marina Verçoza Vianna, Regis Goulart Rosa, Daniel Sganzerla, Thiago Costa Lisboa, Gilberto Friedman

**Affiliations:** 1 Universidade Federal do Rio Porto Alegre RS Brazil Postgraduate Program in Pulmonary Sciences, Universidade Federal do Rio - Porto Alegre (RS), Brazil.; 2 Universidade Federal do Rio Grande do Sul Hospital de Clínicas de Porto Alegre Porto Alegre RS Brazil Hospital de Clínicas de Porto Alegre, Universidade Federal do Rio Grande do Sul - Porto Alegre (RS), Brazil.; 3 Hospital Moinhos de Vento Department of Internal Medicine Porto Alegre RS Brazil Department of Internal Medicine, Hospital Moinhos de Vento - Porto Alegre (RS), Brazil.; 4 Universidade Federal de Ciências de Saúde de Porto Alegre Department of Internal Medicine Porto Alegre RS Brazil Department of Internal Medicine, Universidade Federal de Ciências de Saúde de Porto Alegre - Porto Alegre (RS), Brazil.; 5 Universidade Federal do Rio Grande do Sul Faculdade de Medicina Porto Alegre RS Brazil Faculdade de Medicina, Universidade Federal do Rio Grande do Sul - Porto Alegre (RS), Brazil.

**Keywords:** Critical illness, Respiration, artificial, Long-term care, Survival, Intensive care units

## Abstract

**Background:**

There are notable gaps in the understanding of the underlying pathophysiology of persistent critical illness (PerCI) and its extensive implications for patient outcomes. In this context, whether different PerCI definitions could yield distinct long-term outcomes for intensive care unit survivors is currently unknown.

**Methods:**

This prospective cohort study spanned 10 Brazilian hospitals from March 2015 to December 2017. We enrolled emergency medical and surgical patients with intensive care unit stays exceeding 72 hours and tracked them for more than 12 months after intensive care unit discharge. Chronic patients were classified using four widely recognized persistent critical illness definitions from the literature: 1) mechanical ventilation > 21 days or tracheostomy for mechanical ventilation weaning; 2) mechanical ventilation duration > 14 days; 3) intensive care unit stay > 10 days; and 4) intensive care unit stay > 8 days accompanied by specific clinical conditions warranting extended intensive care unit care. Additionally, the data were compared to those of survivors who did not meet any of the four persistent critical illness criteria.

**Results:**

The study enrolled 1,616 patients, with 609 (37.7%) fulfilling one or more persistent critical illness definitions. The twelve-month survival rates among persistent critical illness patients varied by definition. At 12 months, patients with PerCI definitions centered on mechanical ventilation duration had markedly lower survival rates than non-persistent critical illness patients did (definition 1: HR: 1.49, 95%CI: 1.10 - 2.02; definition 2: HR: 1.66, 95%CI: 1.20 - 2.30). In contrast, definitions based on intensive care unit length of stay produced survival rates more aligned with non-persistent critical illness patients (definition 3: HR: 1.01, 95%CI: 0.82 - 1.25; definition 4: HR: 1.10, 95%CI: 0.88 - 1.30).

**Conclusion:**

Compared with other critically ill patients, patients with persistent critical illness definitions that are based on the duration of mechanical ventilation are associated with reduced 12-month survival, highlighting the impact of prolonged respiratory support on patient outcomes.

## INTRODUCTION

The survival of critically ill patients has improved in recent years;^(1-3)^ however, some of these survivors exhibit a protracted clinical course, marked by persistent organ dysfunction and extended hospital stays. This subset of patients, identified as persistent critically ill (PerCI) patients, constitutes 5 - 20% of all intensive care unit (ICU) admissions and accounts for 20 - 55% of ICU bed occupancy. Furthermore, PerCI patients drive substantial health care costs, with annual expenses in the U.S. estimated at approximately $25 - 35 billion.^(4)^ Most studies indicate that PerCI patients have higher hospital mortality rates than other critically ill patients do.^(5-7)^ However, approximately 60% of PerCI patients are discharged from the hospital but continue to require intensive health care resources postdischarge.^(4,5,8)^ Although there has been a growing interest in the posthospital outcomes of this group,^(4,7,9-12)^ the heterogeneity in PerCI definitions limits comparative outcome analyses.

The concept of PerCI dates back to 1985, when Girard et al. first coined the term ‘chronic illness patients’ to refer to patients who survived an initial episode of critical illness but remained dependent on intensive care.^(13)^ In 2005, an expert panel proposed defining the PerCI by the need for mechanical ventilation (MV) for more than 21 consecutive days at more than 6 hours/day.^(14)^ Over time, with the optimization of MV weaning protocols and early identification of PerCI patients, the ventilation time was reduced to 10 to 14 days.^(4,7,9,10,15)^ In 2014, Medicare and Medicaid formulated their own PerCI criteria: an ICU stay longer than 8 days alongside specific clinical conditions justifying extended care.^(16)^ This concept was introduced with the primary goal of redirecting therapeutic decisions, with transfer to long-term acute care services and a reduction in costs. In 2016, Iwashyna et al., on the basis of the pathophysiological understanding of PerCI, removed the MV-based definition from their definition of PerCI.^(8,17)^ A large-scale cohort study with over 1 million patients revealed that the transition from acute to chronic critical illness occurs around the 10th day of hospitalization,^(18)^ proposing the definition of PerCI as ICU dependence that exceeds 10 days.^(19)^

The focus on MV duration and ICU length of stay as key definitions of PerCI stems from their clinical implications. The duration of MV is closely linked to prolonged critical illness because extended respiratory support often signifies severe underlying pathology and a greater likelihood of post-ICU complications, including ventilator-associated infections and persistent organ dysfunction. The ICU length of stay, on the other hand, serves as a broader measure of ICU dependence and resource utilization. Patients who remain in the ICU for extended periods often face complex, multifactorial illnesses, requiring ongoing critical care support for both respiratory and nonrespiratory conditions.

These criteria - MV duration and ICU length of stay - are therefore integral to clinical decision-making. Prolonged MV is a direct indicator of respiratory failure severity and influences weaning strategies, whereas the length of ICU stay reflects both acute care needs and the potential for PerCI. Together, these measures help clinicians identify patients who are at high risk of long-term morbidity and mortality and who may benefit from specialized post-ICU care or rehabilitation services. Although the term ‘chronic critical illness’ has been the most frequently used term in the literature,^(20)^ we adopted the term PerCI, which encompasses the broader set of criteria found in the literature. Our study addressed key gaps in post-ICU care by providing comparative insights into how these definitions of PerCI affect long-term survival. The comparison of the various PerCI definitions offers a more nuanced understanding of which patients are most vulnerable to poor outcomes and which factors should be prioritized in the allocation of ICU resources and the development of policies aimed at improving post-ICU recovery. Our study sought to compare four primary PerCI definitions, analyzing patient epidemiology and survival over a 12-month period.

## METHODS

We conducted a secondary analysis of a prospective cohort in 10 clinical and surgical ICUs located in the 5 geopolitical regions of Brazil designed to assess long-term survival and disability among general ICU survivors.^(21)^ The study was conducted from March 2015 to December 2017. All surviving patients over 18 years of age who were discharged from the ICUs were consecutively evaluated; patients were approached between 24 and 120 hours after inclusion to request signed consent to participate in the study. Patients who were transferred from another hospital to the ICU; were discharged directly from the ICU to home or to another hospital; refused or withdrew informed content; or had no available telephone contact were excluded.

The study was conducted in accordance with Resolution 466/12 of the Brazilian National Health Council. The protocol was approved by the Research Ethics Committee of the coordinating site (CAAE 04258312.4.1001.5330) and all participating institutions. Informed consent was obtained from all patients or their proxies.

To evaluate the epidemiological profile of PerCI definitions and their effect on 1-year survival, we classified the surviving patients in our cohort into 5 groups: four PerCI groups using the 4 relevant definitions and one comparative group of acute critically ill patients (ICU stay < 72 hours). Patients with ICU stays shorter than 72 hours were excluded to avoid capturing those admitted for short-term, transient critical illnesses, such as postoperative monitoring or minor acute illnesses, which do not typically evolve into PerCIs (Table 1).

**Table 1:** Classification of patients according to study definitions

Groups	Definition 1	Definition 2	Definition 3	Definition 4	Acute critically ill patients
Authors	MacIntyre et al.^(14)^	Iwashyna et al.^(8)^	Darvall et al.^(19)^	Kandilov et al.^(16)^	Robinson et al.^(21)^
MV time	> 21 days or tracheostomy for weaning	> 14 days	Independent	Independent	Independent
ICU length of stay	Independent	Independent	> 10 days	> 8 days	> 72 hours for clinical admission or surgical emergency and > 120 hours for elective surgery
Clinical condition	MV > 21 days	MV > 14 days	Independent	1 out of 5 conditions: Prolonged MV2* Sepsis† Extensive injuries‡ MODS§	Do not present criteria for PerCI

MV - mechanical ventilation; ICU - intensive care unit; MODS - multiple organ dysfunction syndrome; PerCI - persistent critical illness.

* Mechanical ventilation for at least 96 hours or tracheostomy for weaning;

† sepsis or severe infection, including postsurgical infection;

‡ includes pressure ulcer stage III or IV and extensive burns;

§ multiple organ dysfunction syndrome or acute neurological event (ischemic or hemorrhagic stroke and head trauma). Must include ≥ 2 dysfunctional organs (acute or chronic): kidney, heart, lungs, and liver.

The following variables were evaluated to determine the epidemiological profile of patients: (a) sociodemographic characteristics (age, sex, education level, and family income); (b) pre-ICU health status: comorbidities according to the Charlson comorbidity index^(22)^ and previous functional dependence (Barthel index)^(23)^ in the 3 months prior to admission; (c) characteristics of acute critical illness: type of admission to the ICU, severity score of critical illness measured through the Simplified Acute Physiology Score (SAPS) 3^(24)^ or Acute Physiology and Chronic Health Evaluation (APACHE) II^(25)^ depending on the score used by the institution, diagnosis of sepsis (according to the sepsis-II definition),^(26)^ number of organ dysfunctions, delirium, ICU-acquired infection,^(27)^ and length of stay in the ICU and in the hospital.

### Outcomes

We evaluated the 12-month survival of the included patients. In-hospital mortality was defined as the time from ICU discharge to hospital discharge. Follow-up data were obtained through telephone interviews conducted at 3, 6, and 12 months after the ICU discharge date. Death data were obtained through the review of death certificates and medical records. Further protocol details are available in the publication of the original cohort.^(21)^

### Statistical analysis

The sample size of the original cohort was 1500 individuals, which was determined by the number of participants needed to estimate the prevalence of study outcomes. This sample size was increased by 10% because of the multicenter nature of the study and the possibility of different outcomes among services.^(21)^ Categorical variables are described in terms of absolute and relative frequencies, whereas continuous variables are described in terms of means and standard deviations or medians and interquartile ranges, depending on the distribution of the variable. Pearson's chi-square test was used for categorical variables, whereas Student's t test or the Wilcoxon–Mann–Whitney test was used for continuous variables. Regression models were used to evaluate the relationship between independent variables and outcomes, adapting the probability distribution of the outcome of interest in the class of generalized linear models. Survival time is displayed with survival curves and was analyzed using the Cox frailty model. Mortality was adjusted for the following variables: age, Charlson comorbidity index, risk of death (severity score), and preexisting functional dependency. Comparisons between chronic critical illness groups were evaluated by generalized estimating equations, considering that the same patient may be part of more than one group. The generalized estimation equation accommodates correlated outcomes for the same individuals classified into different groups. This method allowed us to assess the impact of each PerCI definition on survival while controlling for the fact that some patients were included in more than one group. The significance level adopted for all comparisons was 0.05. The analyses were performed using R software version 3.4.4.

## RESULTS

A total of 1,616 patients were enrolled in the study until December 2017, with follow-up completed in December 2018. Three hundred fifty-six (22%) patients died before the 12-month follow-up telephone interview, and 121 (7%) patients were lost to follow-up. With respect to critical illness, 68% of patients were admitted to the ICU for medical reasons, and 32% were admitted for surgery. The median risk of death at ICU admission was 14%; 47% of patients needed MV, and 51% needed vasopressors. The median lengths of ICU stay and hospital stay were 6 days and 21 days, respectively.^(28)^

**Figure 1:**
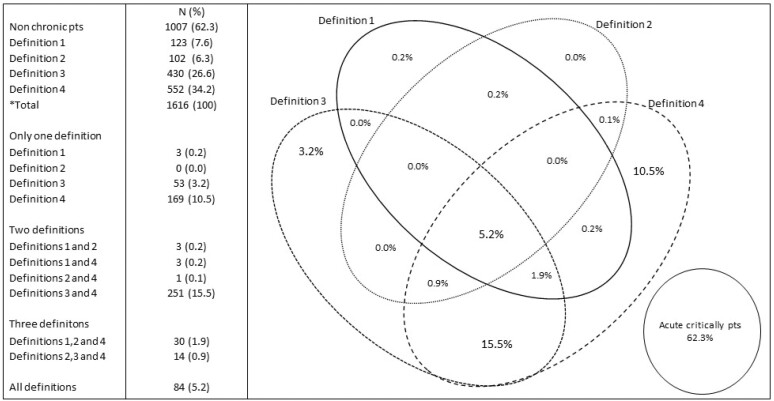
Distribution of patients between study groups (Venn diagram).

Among these patients, 609 (37.7%) patients were classified into one of the 4 PerCI definitions used, and 1007 (62.3%) patients were in the acute critically ill patient group (Figure 1). Eighty-four patients (5.2%) met all 4 PerCI definitions. This number represents 82.3% of definition 1 members, 68.3% of definition 2, 19.5% of definition 3, and 15% of definition 4.

* The sum of the percentages referring to the definitions is greater than 100 because each definition can fit into another.

The time-based PerCI definitions (definitions 1 and 2) group included 222 patients, or 36.5% of the PerCI group. The definitions based on ICU stay time (definitions 3 and 4) group encompassed 603 patients, or 99% of the total PerCI group. A total of 251 patients were included in both the time-based PerCI definitions and PerCI definitions based on ICU stay time groups, representing 58% of the definition 3 patients and 45% of the definition 4 patients.

Table 2 shows the pre-ICU admission status of the patients in the study groups. The groups were similar in terms of preadmission characteristics, except for the acute critically ill patient group, which was older (p < 0.001), had a higher level of education (p = 0.031), and had a higher family income (p = 0.013).

**Table 2:** Characteristics of patients prior to intensive care unit admission

Variables		Definition 1 (n = 123)	Definition 2 (n = 102)	Definition 3 (n = 430)	Definition 4 (n = 552)	Acute critically ill patients (n = 1,007)	p value
Sociodemographic characteristics							
	Age (years)	65 (42 - 75) **A**	64 (46 - 73) **A**	63 (47 - 75) **A**	63 (48 - 75) **A**	68 (56 - 79) **B**	< 0.001
	Age ≥ 65 years	62 (50) **A**	50 (49) **A**	206 (48) **A**	266 (48) **A**	576 (57) **B**	< 0.001
	Female sex	52 (42)	47 (46)	198 (46)	259 (47)	576 (48)	0.330
	University degree	19 (15)	20 (20) **B**	84 (20) **AB**	109 (20) **AB**	231 (23) **AB**	0.031
	House income per capita (U$D)	443 (247 - 756) **A**	461 (296 - 752) **A**	484 (266 - 1041) **A**	487 (269 - 1076) **A**	554 (343 - 1482) **B**	0.013
Health status before ICU							
	Charlson comorbidity index	2 (0 - 3)	2 (0 - 3)	2 (0 - 3)	2 (0 - 3)	2 (0 - 3)	0.63
	Charlson comorbidity index ≥ 2	65 (53)	59 (58)	242 (56)	312 (57)	550 (55)	0,55
	Barthel index	100 (82 - 100)	95 (85 - 100)	100 (85 - 100)	100 (85 - 100)	100 (85 - 100)	0.9
	Physical independence	63 (51)	49 (48)	230 (53)	298 (54)	534 (53)	
	Physical dependence	26 (21)	18 (18)	93 (22)	120 (22)	187 (19)	0.5

ICU - intensive care unit; IU$D - American dollar. The letters A and B in the table indicate groups with similar results (same letter) or statistically significant differences between results (different letters). The results are expressed as the median (interquartile range) or n (%).

Table 3 describes the characteristics of the PerCI patients. The PerCI defined by time on MV (definitions 1 and 2) group had higher severity scores on admission, more organic dysfunction, higher rates of acquired infections in the ICU, and longer stays in the ICU and hospital than did the PerCI defined by ICU stay time (definitions 3 and 4) group. Compared with acute critically ill patients, PerCI patients had higher severity scores on admission and initial diagnosis of sepsis, as well as more organic dysfunction, acquired infection in the ICU, and ICU and hospital stays. The acute patients underwent a greater number of elective surgeries.

**Table 3:** Characteristics of patients in relation to critical illness

		Definition 1 (n = 123)	Definition 2 (n = 102)	Definition 3 (n = 430)	Definition 4 (n = 552)	Acute critically ill patients (n = 1007)	p value
Type of ICU admission							
	Clinical	88 (71)	77 (75)	316 (73)	406 (74)	676 (67)	NS
	Elective surgery	15 (12) **A**	11 (11) **A**	58 (13) **A**	69 (12)**A**	203 (20) **B**	0.002
	Emergency surgery	20 (16)	14 (14)	56 (13)	77 (14)	128 (13)	NS
	Risk of death on ICU admission (%)	36 (21 - 50) **C**	35 (23 - 50) **C**	26 (16 - 46) **B**	26 (16 - 46) **B**	14.6 (10 - 29) **A**	< 0.001
	Sepsis on admission	55 (45) **B**	53 (52) **C**	199 (46) **BC**	280 (51) **BC**	242 (24) **A**	< 0.001
Characteristics of critical illness during ICU stay							
	Number of organic dysfunctions	3 (2 - 4) **C**	3 (2 - 4) **C**	2 (2 - 3) **B**	2 (2 - 3) **B**	1(0 - 2) **A**	< 0.001
	Mechanical ventilation	123 (100) **C**	102 (100) **C**	336 (78) **B**	435 (79) **B**	351 (35) **A**	< 0.001
	Vasopressor	98 (80) **B**	91 (89) **C**	322 (75) **B**	418 (76) **B**	426 (42)) **A**	< 0.001
	Dialysis	28 (23) **BC**	29 (28) **D**	88 (20) **B**	124 (22) **CD**	70 (7) **A**	< 0.001
	Parenteral nutrition	9 (7) **B**	8 (8) **B**	42 (10) **B**	46 (8) **B**	35 (4) **A**	< 0.001
	Transfusion of blood products	39 (32) **B**	42 (41) **C**	123 (29) **B**	144 (26) **B**	130 (13) **A**	< 0.001
	Delirium	48 (39) **B**	50 (49) **C**	172 (40) **BC**	219 (40) **BC**	176 (18) **A**	< 0.001
	ICU acquired infection	89 (72) **D**	80 (78) **D**	169 (39) **C**	185 (33) **B**	35 (3.5) **A**	< 0.001
	ICU length of stay (days)	27 (19 - 38) **D**	30 (23 - 42) **D**	17 (13 - 24) **C**	14 (10 - 21) **B**	5 (4 - 6) **A**	< 0.001
	Hospital length of stay (days)	66 (43 - 89) **D**	68 (44 - 93) **D**	44 (29 - 66) **C**	39 (25 - 60) **B**	20 (13 - 34) **A**	< 0.001

ICU - intensive care unit; NS - not significant. The letters A, B, C and D in the table indicate groups with similar results (same letter) or statistically significant differences between results (different letters). The results are expressed as n (%) or medians (interquartile ranges).

### Hospital mortality and 12-month survival

**Figure 2:**
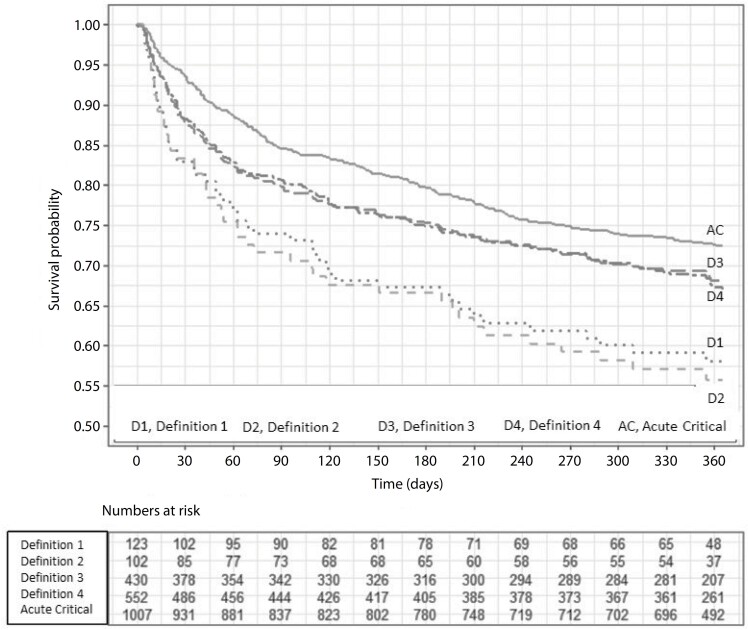
Study group survival curves - Kaplan–Meier.

**Figure 3:**
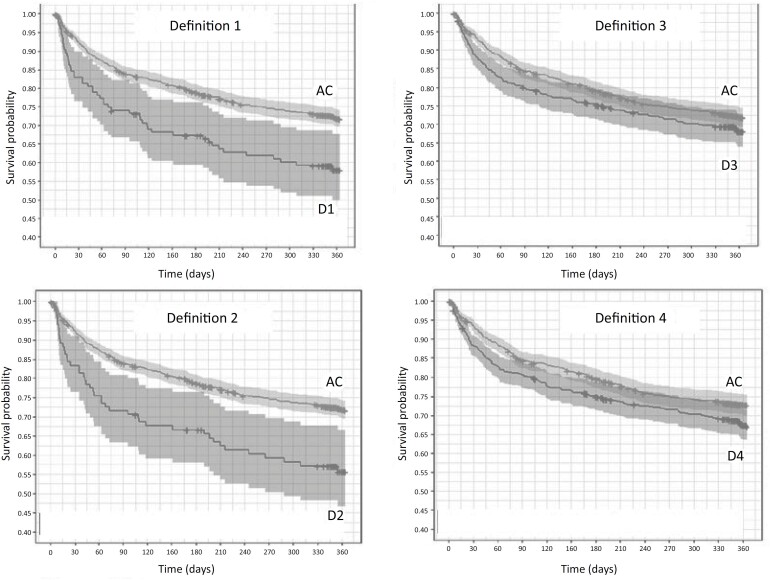
Comparison of Kaplan–Meier survival curves for each definition of chronic critical illness with that of acute critical patients.

Hospital mortality after ICU discharge was 11.1%, with a significant difference between PerCI (14.6%) and acute patients (9%) (adjusted hazard ratio [aHR], 1.56 [95%CI 1.18 - 2.05]; p = 0.002). The overall cumulative mortality rate at 12 months was 28.5%, with 31.3% in the PerCI group and 26.8% in the acute patient group (aHR, 1.07 [95%CI 0.88 - 1.30]; p = 0.47) (Figures 2 and 3).

AC - acute critical.

Table 4 shows the adjusted mortality risk comparisons (age, comorbidities, severity score, and previous functional dependence) between the different PerCI definitions and acute patients at 12 months. The patients with time-based PerCI definitions were associated with higher mortality rates than acute patients were. In contrast, we found no difference between the patients with ICU stay time-based PerCI definitions and acute patients.

**Table 4:** Adjusted mortality risk according to different definitions of persistent critical illness in relation to acute critical illness within 12 months

	Hazard ratio	Confidence interval 95%	p value
Definition 1 (n = 123)*	1.49	1.10 - 2.02	0.012
Definition 2 (n = 102)*	1.66	1.20 - 2.30	0.002
Definition 3 (n = 430)*	1.01	0.82 - 1.25	0.906
Definition 4 (n = 552)*	1.10	0.90 - 1.33	0.344
Any definition (n = 606)†	1.07	0.88 - 1.30	0.469

* To perform the Cox regression, patients classified only in the definition under analysis were used, varying the size of the control group (acute critically ill patients);

† control group for comparison of acute patients, regardless of the definition used (n=1007).

## DISCUSSION

Our study highlights the significant variability in 12-month survival rates depending on the PerCI definition used, particularly those based on MV duration. Compared with definitions based on ICU length of stay (definitions 3 and 4), PerCI definitions based on prolonged MV (definitions 1 and 2) were associated with a greater mortality risk. This association likely reflects the more severe clinical condition of patients requiring extended MV support, as they often experience greater organ dysfunction and secondary complications.

For decades, PerCI has largely been defined as prolonged MV dependence.^(4,6,7,9,10,14,15)^ The removal of MV as a criterion of PerCI by Medicare/Medicaid^(16,29)^ and later by Iwashyna et al..^(8,17)^ has reshaped this concept for its use in public health policies, but the impact of this shift on patient profiles and outcomes remains contentious. Our findings reveal that ICU stay-based definitions encompass a broader patient population, including those with prolonged ICU dependence for nonrespiratory reasons. ICU stay-based definitions allow for earlier identification, but these patients may face different recovery trajectories, leading to more favorable survival outcomes than those requiring extended respiratory support.

The association between definitions linked to prolonged MV and increased mortality may stem from several underlying mechanisms. First, extended MV often reflects a more severe initial illness, leading to prolonged organ dysfunction and a cascade of complications, such as ventilator-associated pneumonia, which can exacerbate patients’ clinical conditions.^(30-32)^ Second, prolonged reliance on MV may impede the recovery of respiratory muscle function, further delaying weaning processes and increasing the risk of respiratory failure postdischarge.^(30)^ Additionally, patients who require long-term MV frequently experience persistent inflammation and immune dysregulation, rendering them vulnerable to secondary infections and comorbidities that can adversely affect survival outcomes.^(31,32)^ The psychological impact of prolonged critical illness, including anxiety and depression, may also contribute to poorer prognosis by hindering rehabilitation efforts. Collectively, these factors highlight the complex interplay between prolonged MV and increased mortality risk, necessitating targeted interventions to improve patient outcomes in this population. Although the impact of interventions, such as protocols for weaning from MV and psychological support, on outcomes after ICU discharge has been explored in various studies, the evidence remains mixed. Interventions may include early mobilization and rehabilitation, weaning protocols, nutritional support and psychological support.^(33-36)^

In settings with limited resources, such as many parts of Brazil and other low- to middle-income countries, the findings of our study could help guide resource allocation and policy development. Patients on prolonged MV often require intensive resources and prolonged ICU stays. Improving ICU discharge planning and implementing follow-up programs may improve outcomes in a cost-effective manner. While our study was conducted on patients in Brazilian ICUs, our findings may have broader relevance to health care systems in other countries, particularly those with similar resource constraints. Additionally, strategies for improving outcomes in MV-dependent patients are likely applicable in a wide range of health care systems. However, differences in ICU admission practices, therapeutic limitations, and access to post-ICU care in other health care systems should be considered when generalizing our results.

Some limitations warrant discussion. First, our decision to exclude patients with ICU stays of less than 72 hours may have influenced the prevalence of PerCI cases in our sample compared with previous studies.^(5,6,8)^ Second, by omitting these short-stay patients, we likely increased the chronicity potential of our cohort, which could skew the acute critically ill patient sample toward a higher surgical risk group. Follow-up studies that evaluated patients from the moment of admission report one-year mortality rates ranging from 40% to 70%.^(4,8-10,15)^ Although extensive data compared mortality between acute and chronic patients, our distinctive approach narrowed the comparative framework. For example, Cox et al. reported similar one-year mortality rates between patients on prolonged MV for more than 21 days and those with shorter durations exceeding 48 hours,^(9)^ and Douglas et al. reported similar survival rates at 12 months for patients with MV durations between 24 and 96 hours and MV durations > 96 hours.^(37)^ Furthermore, while mortality was greater across all PerCI groups, we did not consider therapeutic limitations, which could significantly affect outcomes. These limitations affect the generalizability of our findings, as prior studies have shown varying prevalence rates of PerCI among different patient populations.^(5,6,8)^

Although our study groups were similar in terms of preadmission characteristics, the acutely critically ill group was found to be older, with a higher level of education and family income. These differences may have influenced the observed outcomes. Age is a well-established factor associated with increased mortality and poor recovery in critically ill patients. However, higher education and family income are linked to better access to health care resources and improved post-ICU recovery, which may have mitigated some of the age-related risks in this group. While we adjusted for age in our mortality analysis, the potential influence of socioeconomic factors on survival and recovery remains a limitation.

Finally, it could be more important for clinicians to share their prognostic impressions or expectations of clinical outcomes that consider the impact of post-ICU syndrome on quality of life with families. In addition, variations in post-ICU care, comorbidities, and therapeutic limitations likely influenced survival outcomes in our cohort. Unfortunately, detailed data on postdischarge care, such as access to rehabilitation, home care, or follow-up clinics, were not available. Therefore, for this analysis, we chose mortality as a difficult but easy-to-follow outcome.

Despite these limitations, our study provides substantial insights. Although the term ‘chronic critical illness’ remains widely used, our study underscores the need for precise definitions in clinical and research settings. The ongoing use of various terms and criteria to describe PerCI emphasizes the complexity of this patient population and the importance of standardized approaches for identifying and managing these patients in critical care settings.

To our knowledge, no studies have contrasted recent PerCI definitions with prolonged MV-based definitions. By profiling PerCI epidemiology and its effects on long-term survival, our findings support informed therapeutic decisions and offer families a clearer outlook on this outcome. Unlike prior studies, we compared PerCI patients with patients under intermediate-intensity care, suggesting that this group has a unique outcome distinct from that of short-stay patients. Additionally, we expanded the epidemiological and prognostic understanding of PerCI within developing nations, where the literature remains sparse.

## CONCLUSION

Our findings show that persistent critical illness definitions based on intensive care unit stay time present similar one-year survival rates as acute critically ill patients do, excluding patients with short stays in the intensive care unit. In contrast, patients with PerCI definitions based on prolonged mechanical ventilation are associated with lower one-year survival than patients in the other groups in the study are.
